# Generalization—a key challenge for responsible AI in patient-facing clinical applications

**DOI:** 10.1038/s41746-024-01127-3

**Published:** 2024-05-21

**Authors:** Lea Goetz, Nabeel Seedat, Robert Vandersluis, Mihaela van der Schaar

**Affiliations:** 1 Artificial Intelligence and Machine Learning, GSK, London, UK; 2 Department of Applied Mathematics and Theoretical Physics, University of Cambridge, Cambridge, UK; 3 Cambridge Centre for AI in Medicine, University of Cambridge, Cambridge, UK

**Keywords:** Health care, Machine learning

## Abstract

Generalization – the ability of AI systems to apply and/or extrapolate their knowledge to new data which might differ from the original training data – is a major challenge for the effective and responsible implementation of human-centric AI applications. Current debate in bioethics proposes selective prediction as a solution. Here we explore data-based reasons for generalization challenges and look at how selective predictions might be implemented technically, focusing on clinical AI applications in real-world healthcare settings.

Whether in healthcare, finance or education, generalization is a core challenge for real-world impact in all areas of human-centric Artificial Intelligence (AI). There are currently limited technical solutions that work for generalization challenges in patient-facing clinical applications of machine learning (referred to as clinical ML” hereafter). To address this, recent work in bioethics^1^ advocates selective deployment of AI in healthcare and provides a thorough analysis of the ethical implications. “Selective deployment” suggests that algorithms should not be deployed for groups underrepresented in their training datasets due to risks around poor or unpredictable algorithm performance. Here, we use a case study in clinical ML to explore available technical choices for the implementation of selective deployment, with the goal of improving patient outcomes (see Fig. 1).

**Fig. 1:**
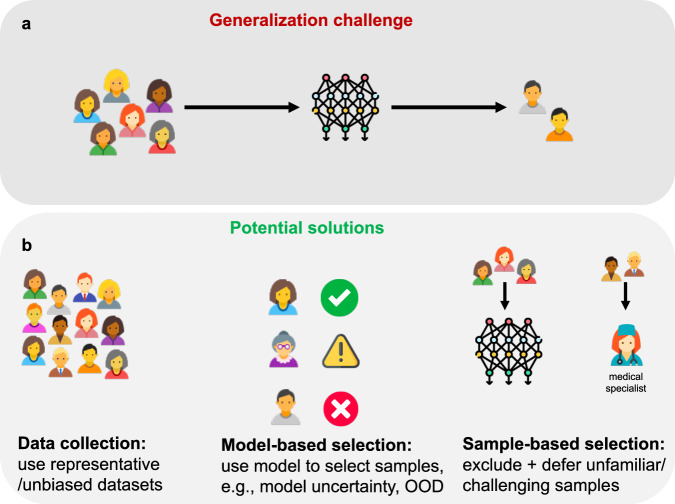
The generalization challenge and potential solutions. **a** ML models trained on biased or non-representative datasets may fail to generalize to a subset of patients. **b** Potential solutions to the generalization challenge (left to right). *Data collection* augments training datasets with additional (real or synthetic) data so models can learn on all patients encountered during deployment. Limitation: data collection might be expensive or logistically challenging. *Model-centric selection* uses an additional ML model, e.g. an out-of-distribution (OOD) detector, or the ML model itself, e.g. model uncertainty, to select samples on which model outputs are trustworthy and to defer others to a clinician. Limitation: reliance on the model performing sample selection and patient exclusion. *Sample-centric selection* excludes samples where untrustworthy model outputs are expected either upfront or during deployment, deferring these samples to clinicians. Limitation: if sample exclusion leads to coverage gaps, it can harm model performance by exacerbating existing biases. Head icons from https://icons8.com/.

Why is generalization a challenge in clinical AI? In short, expressive ML models, especially deep neural networks, are prone to overfitting, i.e., they over rely on low-level features and learn spurious correlations in a dataset, when underspecified^2,3^. Furthermore, training data reflecting societal prejudices or lacking diversity can result in algorithmic biases that can cause models to generalize less well to underrepresented groups. These problems are exacerbated in clinical applications, where datasets are high dimensional, contain the inherent uncertainties of biological systems, are often small and noisy, contain large numbers of missing values, and may not be representative of the target population^4,5^. Furthermore, pre-training for transfer learning, a ML technique that can enable generalization^6^, is often inappropriate in clinical contexts, given the significant difference between small, domain-specific medical datasets and large, general-purpose pre-training datasets like ImageNet.

ML models that do not generalize may fail silently, i.e. perform significantly worse on new samples or individuals unnoticed, especially if not externally validated^7^. Ignoring these challenges and applying ML models in the clinic regardless is irresponsible as it may harm patients from underrepresented groups.

## Case study

Breast cancer predominantly affects biological women with a 100:1 ratio compared to biological men^8^. Consequently, men experience substantially worse health outcomes^9^, and are underrepresented in clinical datasets. Differences in disease etiology make it challenging for predictive algorithms trained on data from biologically female to generalize to biological males. For instance, a recent breast cancer prognostic algorithm^10^, trained only on female data, offers accurate predictions for women but is expected to underperform for biological men due to exclusion from the dataset. Excluding men from using this algorithm safeguards them from potentially unreliable predictions, but raises ethical concerns about fairness and equal access to advanced treatments. We could achieve equality between men and women by “leveling down”^11^ the standard of care for women to that of men, e.g., by reducing the accuracy of predictions for women; or withholding the algorithm completely. An emerging discussion in bioethics^1^ considers withholding best-in-class treatment from women only to achieve fairness across sexes unethical. It is argued that men’s standard of care would not worsen if the prognostic algorithm became available to women. Deploying these algorithms for women could, in fact, enhance overall breast cancer research and AI development. Due to the prevalence of breast cancer and data limitations, they recommend selective deployment of such algorithms for responsible and effective use.

While biological variances such as sex provide a rationale for selective deployment in examples such as our breast cancer case study, selective deployment based on sociocultural factors such as gender presents a more complex issue which we explore below (see *Ethical considerations*).

### What is “responsible AI” in clinical applications?

As in the breast cancer algorithm example, calls for strict fairness may not necessarily be a responsible approach in clinical AI. Instead, the criterion for responsible use of ML is whether we can *trust the predictions of a model*. For brevity and clarity of argument, we focus our discussion on predictive models; however, the same arguments equally apply to other ML algorithms, such as unsupervised or generative models. First, we need to be able to trust that a model produces accurate predictions on any given patient’s input data. For this, input data during deployment should be similar to the training, validation and test datasets where the model is validated and is shown to perform reliably. Second, where inputs during deployment are drawn from a different data distribution, or where they are ambiguous or inherently difficult, we need the model to “know what it doesn’t know”^12^.

*In summary: to trust model predictions, we need to identify the samples – individuals, subgroups and features – on which the model performs well, deferring others to complementary approaches, to prevent model failures and potential harm to patients*^13^. Notably, the subgroups on which we cannot trust model predictions may not align with traditional patient stratifications such as sex, age or ethnicity and may often be intersectional.

### Selecting samples—current practice

If the exclusion of samples upfront for training—as in our case study—seems extreme, this is in fact common practice in the ML and healthcare communities, but it is currently largely performed ad hoc and supported by little documentation or principled reasoning. For example, clinical trials for triple-negative breast cancer often exclude patients who are HER2-negative and have low estrogen receptor expression, even though they represent a significant proportion of breast cancer cases (15% in the United States)^14^. Another common practice is to exclude samples with “too many” missing values, which may or may not be missing at random. This often depends on the context and individual researcher discretion, so cutoff values greatly vary between studies. This motivates the usage of principled, quantitative algorithmic selection approaches, which have been shown to improve model performance^15^.

### Algorithmic selection of samples for trustworthy predictions

**Fig. 2:**
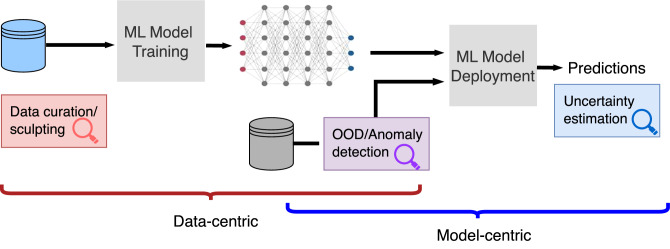
Sample selection underpins trustworthy predictions. Sample selection can be achieved by data-centric methods of data curation/sculpting before training the model, or model-centric sample deferral with uncertainty estimation. Out-of-distribution and anomaly detection methods lie at the intersection, wherein we flag samples preemptively.

Broadly, we can distinguish between sample/data-centric and model-centric methods for selecting the samples on which we can trust model predictions, as outlined in Fig. 2.

*Sample/data-centric methods*—i.e. data curation, or data sculpting^15^—aim to quantify the value and importance of individual samples and filter out samples before model training. For example, this could mean removing samples from genome-wide association studies, where uncertainty in polygenic risk score estimates for individuals can have a large impact on subsequent analyses^16^, or removing samples from clinical datasets due to poor quality (artifacts/measurement errors) or bias at the individual sample level^17^. By removing noisy or mislabeled samples from a training dataset, model training and performance can be increased for the remaining samples^18^. For examples and methods, see^4,15^. Data curation by rejecting samples that do not match the curation criteria during inference is the most stringent way to prevent untrustworthy model predictions. Where appropriate given other considerations of utility, fairness and justice, this approach could be applied in the high-risk scenario of clinical AI, where biases in the training data, coupled with subsequent inaccurate model predictions, could have serious negative consequences for individual patients. These sample- or data-centric methods could further be supplemented with the model-centric methods described next.

*Model-centric methods* – to be trustworthy, it is crucial for models to indicate when predictions for samples are likely to be incorrect, i.e. we desire estimates of how uncertain a model is for any given prediction. This is especially relevant for deep neural networks, which can provide overconfident point estimates. Uncertainty estimation provides a principled solution and can roughly be grouped into Bayesian or approximate-Bayesian, model distillation and ensemble-based methods^19^, with the estimates often decomposed into uncertainty arising from the model (epistemic uncertainty) and uncertainty inherent in the data (aleatoric uncertainty). Another orthogonal strand of approaches is conformal prediction^20^, which produces prediction intervals with coverage guarantees, where the interval size reflects uncertainty. Irrespective of how model uncertainty is estimated, typically an uncertainty threshold is required, below which predictions are considered too unreliable and therefore untrustworthy. Uncertain model predictions most likely arise for samples that do not match the training data distribution or that exhibit in-distribution inconsistency due to low coverage. These samples can also directly be flagged with methods from the related fields of anomaly or novelty detection, open-set recognition and out-of-distribution (OOD) detection^21^.

It is worth noting that any of the aforementioned technical approaches to sample selection may have similar failure modes to the predictive model itself: if they do not generalize well, they will not provide the required safeguarding for exactly those samples for which it is most needed. We therefore recommend not exclusively relying on model-centric methods in medium- and high-risk clinical applications, but to always involve a human-in-the-loop where the outcome directly impacts individual patients.

### Ethical considerations

Proactive sample selection for trustworthy model predictions could be problematic if it would consistently exclude/defer individuals from already marginalized subgroups. In particular, consider selective deployment not based on biological determinants but instead on socio-cultural constructs. In this case, proactive sample selection might unintentionally be grounded in historical marginalization and could therefore propagate injustice, rather than accounting for justified biological variances. Crucially, where biological and socio-cultural concepts interact, socio-cultural factors must be considered carefully even when selecting samples based on biological determinants. The distinction between biological determinants and socio-cultural constructs is important because individuals from marginalized subgroups are more likely to be underrepresented or missing from datasets used for model training, thus exacerbating existing social and health inequalities. The literature considers broadly three options to address this issue, which we summarize; for a more in-depth ethical analysis, see^1^.

*Option 1:* Delay deployment until algorithms work equally well for all, avoiding harm but delaying benefits for those where current models are accurate.

*Option 2*: Expedite deployment, ignoring generalization issues, risking harm to underrepresented groups.

*Option 3*: Selectively deploy, using algorithms where safe and deferring others to human medical professionals.

Options 1 and 2 pose ethical issues. Delaying deployment until achieving equitable performance across all subgroups might not be practically feasible and could needlessly harm or “level down” health outcomes to those who could be helped now, while indiscriminately expediting deployment risks harming underrepresented patients. Unfortunately, with pressure to bring ML to the clinic to improve efficiency and patient outcomes, there are already examples of indiscriminate deployment that may harm minority groups^22^. Option 3, selective deployment is a potentially contentious option emerging in the bioethics literature^1^ which, under circumstances where the withholding of deployment or indiscriminate deployment are considered unethical, could serve as an intermediary solution until equal performance can be reached across all subgroups. Importantly, selective deployment must be balanced with a commitment to equity: any potential discriminatory consequences must be proactively mitigated, for example, individuals excluded over concerns of subpar model performance should be deferred to an expert clinician in order to ensure an equivalent standard of care.

Balancing practicality with equity is a pervasive issue that will only become more pressing as AI is increasingly applied in healthcare settings. Thus, rather than advocating for selective deployment per se or trying to resolve the associated ethical issues, our aim in this work is to highlight generalization challenges as an underlying ML problem, and to make the consideration of this option much more technically informed by pointing to principled algorithmic approaches (see *Algorithmic selection of samples for trustworthy predictions*). Furthermore, we hope to bring the bioethical debate on selective deployment to the ML and healthcare community to start a conversation with a broad range of stakeholders, including patient groups. Although selective deployment has the potential to temporarily maintain health disparities, debate is needed whether in the current data and modeling environment in healthcare, this option may represent the most ethical tradeoff between competing considerations around utility, safety, and equity.

### Future research and moving forward

While we outline approaches for selective prediction when ML models do not generalize, we also encourage future research into generalization in the small sample regime in clinical ML and other areas of human-centric AI. To begin with, we need a better understanding of why domain generalization often does not outperform expected risk minimization. Another promising direction is the use or fine-tuning of large-scale, generalist foundation models on scarce data or exploring training paradigms, such as model distillation or contrastive learning adapted to the low-data regime. Furthermore, synthetic data may improve model generalization, both to augment small datasets during training and for simulating real-world distribution shifts during model evaluation, yet should leverage fair generation approaches e.g. ^23^ to prevent the propagation of biases. Finally, more research is needed on active data-centric AI techniques to guide data collection and valuation, which are essential for equitable deployment of clinical ML.

Putting ML systems into practice takes time, but updating these systems with new data or new models is comparatively straightforward. Thus, we should find ethical ways to deploy ML algorithms in the clinic or other areas of human-centric AI, despite current generalization challenges. We encourage ML researchers to explore sample selection strategies – appropriately matched to the risk level and context of the ML application – as they are looking for ways to make their clinical ML applications trustworthy and safe for all patients.

### Reporting summary

Further information on research design is available in the Nature Research Reporting Summary linked to this article.

### Supplementary information

Reporting Summary
